# MiR-129-3p promotes docetaxel resistance of breast cancer cells via CP110 inhibition

**DOI:** 10.1038/srep15424

**Published:** 2015-10-21

**Authors:** Yuan Zhang, Yu Wang, Yifang Wei, Mengyang Li, Shentong Yu, Mingxiang Ye, Hongmei Zhang, Suning Chen, Wenchao Liu, Jian Zhang

**Affiliations:** 1 The State Key Laboratory of Cancer Biology and Department of Oncology, Xijing Hospital, The Fourth Military Medical University, Xi’an 710032, China; 2 The State Key Laboratory of Cancer Biology and Department of Biochemistry and Molecular Biology, The Fourth Military Medical University, Xi’an 710032, China; 3 Department of Pulmonary Medicine, Xijing Hospital, The Fourth Military Medical University, Xi’an 710032, China; 4 Department of Pharmacy, Xijing Hospital, The Fourth Military Medical University, Xi’an 710032, China

## Abstract

Docetaxel is commonly used as an effective chemotherapeutic agent in breast cancer treatment, but the underlying mechanisms of drug resistance are not fully understood. The purpose of this study was to investigate the possible role of miR-129-3p in breast cancer cell resistance to docetaxel. MiR-129 and miR-129-3p inhibitor were transfected into breast cancer cells to investigate their effects on chemoresistance to docetaxel. The function of miR-129-3p was evaluated by apoptosis, cell proliferation, and cell cycle assays. We found that miR-129-3p was up-regulated in MDA-MB-231/Doc cells, concurrent with CP110 down-regulation, compared to the parental MDA-MB-231 cells. *In vitro* drug sensitivity assays demonstrated that miR-129-3p inhibition sensitized MDA-MB-231/Doc and MCF-7 cells to docetaxel, whereas miR-129 overexpression enhanced MDA-MB-231 and MCF-7 cell resistance to docetaxel. Ectopic miR-129 expression reduced CP110 expression and the luciferase activity of a CP110 3′ untranslated region-based reporter construct in MDA-MB-231 cells, suggesting that CP110 is a direct miR-129-3p target. We demonstrated that restoration of CP110 expression in MDA-MB-231 and MCF-7 cells by miR-129 overexpression rendered the cells sensitive to docetaxel. In a nude xenograft model, miR-129 up-regulation significantly decreased MDA-MB-231 cells’ response to docetaxel. Our findings suggest that miR-129-3p down-regulation potentially sensitizes breast cancer cells to docetaxel treatment.

Breast cancer is one of the most common cancer types and the main cause of cancer death in women worldwide^1^. In China, breast cancer accounts for approximately 16% of the ten most common cancers in females, and the number of new breast cancer cases has been increasing each year^2^. Chemotherapy is an important component in breast cancer treatment and often involves the administration of anthracyclines together with taxanes. Docetaxel belongs to the taxane class of chemotherapeutic agents and is very important in chemotherapy treatment of a variety of malignancies, including breast cancer^3^,^4^. Microtubules are very important in a number of cellular processes, which include maintenance of cellular shape, intracellular vesicle transport, and cell division^5^,^6^. Docetaxel exerts its effects by binding to β-tubulin, which is one of the major components of microtubule, resulting in preventing the depolymerisation of microtubules and the growth arrest of tumor cells at the G2-M phase^7^. However, many breast patients are intrinsically resistant or acquire resistance during the course of docetaxel treatment, which leads to recurrence and metastasis. To date, there are no clinically useful predictive factors to distinguish patients who are likely to respond to docetaxel treatment.

Although the resistance mechanism to docetaxel is still unclear, several preliminary and unconfirmed observations have been reported^8^,^9^,^10^,^11^. MicroRNAs (miRNAs) are a group of small (~22 bp), non-coding, single-stranded RNA molecules that have posttranscriptional regulatory roles in physiological and pathological processes^12^,^13^,^14^,^15^,^16^. MiRNAs bind the 3′ UTR of target gene mRNAs, resulting in mRNA destabilization and translational repression. Recently, many studies have shown that miRNAs are broadly involved in cancer development and drug resistance^17^,^18^,^19^,^20^,^21^. MiR-129 expression has been shown to be involved in the progression of several types of cancers including breast cancer, and its expression has been shown to correlate with patient survival^22^,^23^,^24^,^25^,^26^,^27^. Furthermore, a recent study indicated that miR-129 promoted apoptosis and enhanced chemosensitivity to 5-fluorouracil in colorectal cancer^28^. Both of the mature miRNAs, miR-129-3p and miR-129-5p, originate from opposite arms of the same precursor miR-129, and miR-129-3p is the main mature miRNA formed from this precursor^29^. This preponderance of miR-129-3 might be due to its involvement in other biological activities or to degradation of miR-129-5p^30^,^31^. The direct miR-129 target CP110 is a conserved centriolar protein known to suppress ciliogenesis^32^,^33^,^34^. In addition, previous studies indicated that CP110 plays an essential role in centrosome duplication, and its deregulation may contribute to genomic instability^35^,^36^. However, to the best of our knowledge, no studies have focused on the association of miR-129-3p dysregulation with breast cancer cell resistance to docetaxel.

In this study, we developed docetaxel-resistant cell clones (MDA-MB-231/Doc) from the human breast cancer cell line MDA-MB-231. The results indicated that miR-129-3p was up-regulated in MDA-MB-231/Doc cells compared to their parental cell line MDA-MB-231. We demonstrate for the first time that miR-129-3p confers docetaxel resistance in breast cancer cells, mediated at least in part by targeting CP110.

## Results

### Characterization of docetaxel-resistant breast cancer cells

**Figure 1:**
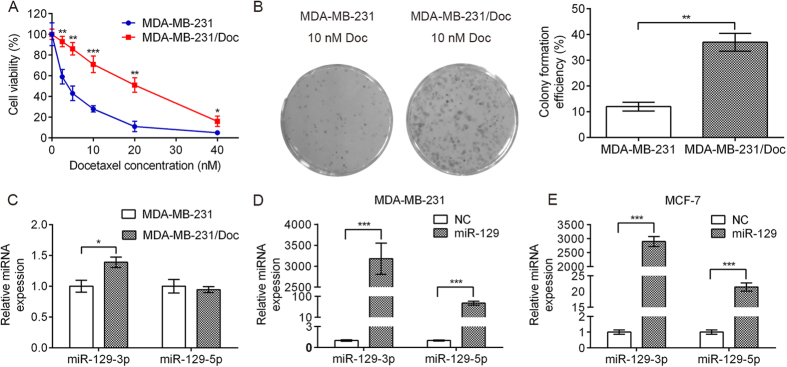
MiR-129-3p is highly expressed in MDA-MB-231/Doc resistant cells and stably transfected cells. (**A**) MTT assay showing that MDA-MB-231/Doc cells were more resistant to docetaxel than MDA-MB-231 cells. (**B**) Colony formation assay revealed a significant increase in proliferative ability of MDA-MB-231/Doc cells treated with 10 nM docetaxel. (**C**) Quantitative real-time PCR analysis of miR-129-3p and miR-129-5p expression in parental MDA-MB-231 cells compared to docetaxel-resistant MDA-MB-231/Doc cells. (**D**,**E**) Quantitative real-time PCR analysis of miR-129-3p and miR-129-5p expression in MDA-MB-231 and MCF-7 cells stably transfected with miR-129. Each bar represents the mean ± SD. p values were calculated using Student’s t-test (*p < 0.05, **p < 0.01, ***p < 0.001).

To develop an *in vitro* model of acquired docetaxel resistance, we continuously exposed the human breast cancer cell line (MDA-MB-231) to 10 nM docetaxel for 8 months until cells had become resistant to docetaxel. The morphology of MDA-MB-231/Doc cells resembled that of the MDA-MB-231 cell line. We determined MDA-MB-231 and MDA-MB-231/Doc cell sensitivity to various concentrations of docetaxel using MTT assays. As shown in Fig. 1A, MDA-MB-231/Doc cells were significantly resistant to docetaxel compared to parental MDA-MB-231 cells. Colony formation assays revealed a significant increase in proliferative ability of MDA-MB-231/Doc cells (Fig. 1B).

### MiR-129-3p expression significantly increased in docetaxel-resistant breast cancer cells

To explore whether miR-129 is involved in breast cancer cell resistance to docetaxel, we evaluated miR-129-3p and miR-129-5p expression in MDA-MB-231, MDA-MB-231/Doc cells, MDA-MB-231 and MCF-7 cells stably transfected with miR-129 using quantitative real-time PCR. We found that miR-129-3p was up-regulated in MDA-MB-231/Doc cells compared to parental MDA-MB-231 cells, whereas miR-129-5p expression did not significantly differ between MDA-MB-231 and MDA-MB-231/Doc cells (Fig. 1C). We also found the significant up-regulation of miR-129-3p, to a greater level than miR-129-5p, in MDA-MB-231 and MCF-7 cells stably transfected with miR-129 compared with parental cells (Fig. 1D,E).

### CP110 is a direct miR-129-3p target in breast cancer cells

**Figure 2:**
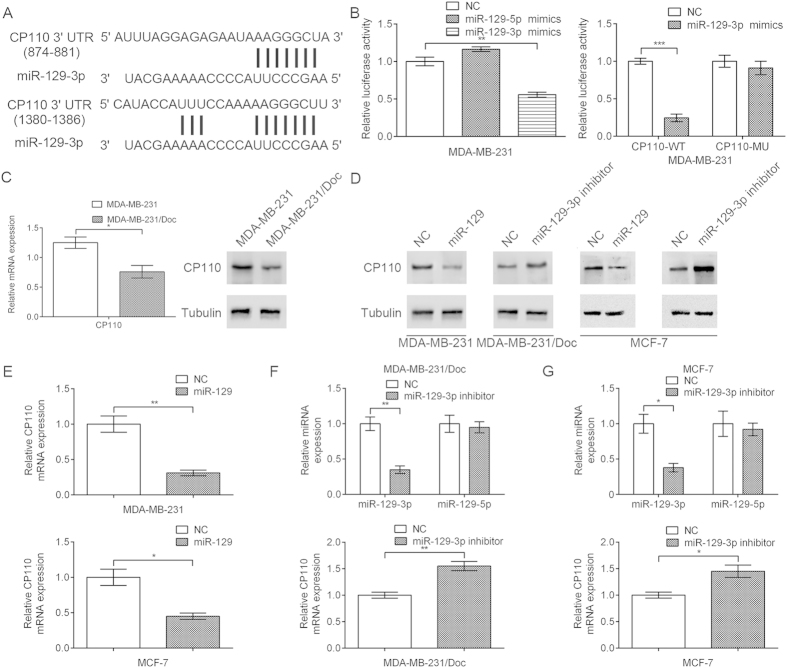
CP110 is a miR-129-3p target. (**A**) The miR-129-3p binding sites in the CP110 3′ UTR are shown, as predicted by bioinformatics analysis. (**B**) miR-129-3p transfection inhibited firefly luciferase activity of pMIR-REPORT-3′-UTR-CP110 (wild-type, wt), and such inhibition was absent when the miR-129-3p-binding site was mutated (mutant, mu). The effect of miR-129-3p on CP110 expression was normalized and compared to the negative miRNA transfection. (**C**) Quantitative real-time PCR and western blot analysis of CP110 mRNA and protein expression in parental MDA-MB-231 cells compared to docetaxel-resistant MDA-MB-231/Doc cells. (**D**) miR-129 overexpression inhibited CP110 protein expression and down-regulation of miR-129-3p facilitated CP110 protein expression. (**E**) Quantitative real-time PCR analysis of CP110 in MDA-MB-231 and MCF-7 cells stably transfected with miR-129. (**F**,**G**) Quantitative real-time PCR analysis of CP110 in MDA-MB-231/Doc and MCF-7 cells transfected with miR-129-3p inhibitor. Each column shows the mean of three independent experiments. Each bar represents the mean ± SD. *p < 0.05, **p < 0.01, ***p < 0.001, Student’s t-test.

To identify genes involved in miR-129-3p-associated docetaxel resistance of breast cancer cells, we searched for putative miR-129-3p targets using both TargetScan and Sanger database searches. We identified two putative miR-129-3p binding sites at positions 874–881 (AAGGGCUA) and 1380–1386 (AAGGGCU) in the CP110 mRNA 3′ UTR region (Fig. 2A), consistent with a previous study^34^. To assess whether miR-129-3p directly regulates CP110 expression through target sites in the 3′ UTR of CP110 mRNA in breast cancer cells, we cloned the CP110 3′ UTR region with wild-type or mutant miR-129-3p binding sites into the pMIR-REPORT vector. Remarkably, we observed a significant decrease in relative luciferase activity in MDA-MB-231 cells when the CP110 3′ UTR was co-transfected with miR-129-3p mimics, but not with control miRNA mimics or miR-129-5p mimics (Fig. 2B, left). In addition, the luciferase activity of the wild-type CP110 3′ UTR reporter, but not that of the mutant, decreased in MDA-MB-231 cells transfected with miR-129-3p mimics, suggesting that the predicted binding sites were indeed miR-129-3p binding regions within the CP110 3′ UTR (Fig. 2B, right). A point mutation resulted in the change of AGGGCT→ACCCGT in the CP110 3′ UTR. As expected, this suppression was abolished by deletion of part of the perfectly complementary sequence in the CP110 3′ UTR, which disrupted the interaction between miR-129-3p and CP110. Furthermore, quantitative real-time PCR and western blots showed that CP110 levels were significantly lower in MDA-MB-231/Doc cells than in MDA-MB-231 cells (Fig. 2C). To further ascertain whether miR-129-3p regulates CP110, we transfected MDA-MB-231 cells, MDA-MB-231/Doc cells, and MCF-7 cells with miR-129 or a miR-129-3p inhibitor. We found that miR-129 overexpression reduced CP110 expression at both the mRNA and protein level, whereas transfection of the miR-129-3p inhibitor increased CP110 expression in breast cancer cells (Fig. 2D–G). Taken together, these results strongly suggested that CP110 is a direct miR-129-3p target in the breast cancer cells.

### MiR-129-3p overexpression and CP110 repression promote docetaxel resistance of breast cancer cells

**Figure 3:**
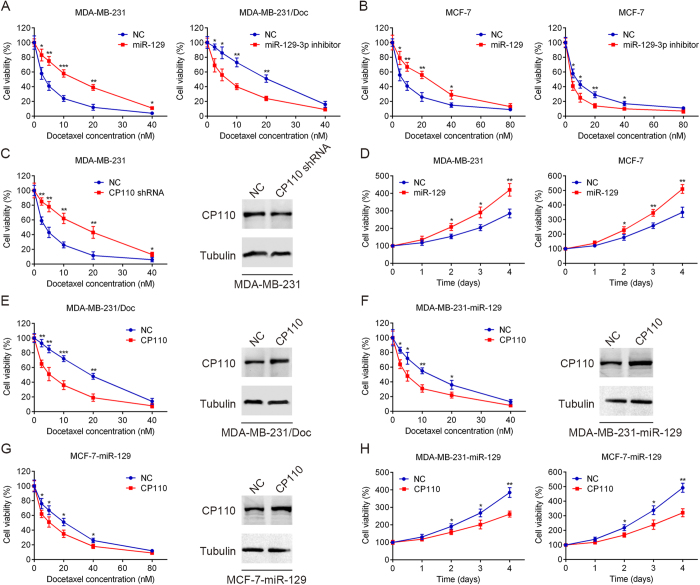
MiR-129-3p and CP110 are functionally involved in the docetaxel response of breast cancer cells. (**A**,**B**) MDA-MB-231, MDA-MB-231/Doc, and MCF-7 cells were transfected with miR-129 or miR-129-3p inhibitor for 48 hrs and then treated with various concentrations of docetaxel for 48 hrs, and cell viability was analyzed using MTT assay. (**C**) MDA-MB-231 cells were transfected with CP110 shRNA or NC for 48 hrs, then treated with various concentrations of docetaxel for 48 hrs, and cell viability was assessed using MTT assay. Transfection efficiency was assessed by western blot analysis. (**D**) Time-dependent effects of miR-129 on the proliferation of MDA-MB-231 and MCF-7 cells were confirmed using MTT assay, following treatment with docetaxel. (**E**–**G**) MDA-MB-231/Doc, MDA-MB-231-miR-129 and MCF-7-miR-129 cells were transfected with CP110 or NC for 48 hrs, then various concentrations of docetaxel were added for 48 hrs, and cell viability was assessed using MTT assay. Transfection efficiency was assessed by western blot analysis. (**H**) Time-dependent effects of miR-129 on the proliferation of MDA-MB-231-miR-129 and MCF-7-miR-129 cells were confirmed using MTT assay, following treatment with docetaxel. Data are presented as the mean ± SD. *p < 0.05, **p < 0.01, ***p < 0.001 by Student’s t-test.

Docetaxel-resistant MDA-MB-231/Doc cells expressed higher miR-129-3p levels and lower CP110 levels than parental MDA-MB-231 cells. To assess whether miR-129-3p expression affected docetaxel sensitivity, we transfected MDA-MB-231 cells, MDA-MB-231/Doc cells, and MCF-7 cells with miR-129 or the miR-129-3p inhibitor. Transfected cells were exposed to various concentrations of docetaxel for 48 hrs and assessed via MTT assay. As shown in Fig. 3A,B, miR-129 transfection promoted MDA-MB-231 and MCF-7 cell resistance to docetaxel treatment. In contrast, miR-129-3p inhibitor transfection increased docetaxel sensitivity of MDA-MB-231/Doc and MCF-7 cells. To further explore whether CP110 affected cell sensitivity to docetaxel, we silenced CP110 expression in MDA-MB-231 cells by shRNA and measured cell sensitivity to docetaxel treatment. Knockdown of CP110 increased MDA-MB-231 cells resistance to docetaxel treatment compared to the control (Fig. 3C). The transfection efficiency was detected by western blot analysis. In addition, we constructed a concentration-dependent curve based on the cell viability of MDA-MB-231 and MCF-7 cells treated with docetaxel alone or with miR-129 and docetaxel combination (Fig. 3D). Our data showed that miR-129 overexpression and CP110 repression could significantly increase the resistance of breast cancer cells to docetaxel treatment.

### Restoration of CP110 re-sensitized breast cancer cells to docetaxel

To further determine whether the resistance of breast cancer cells to docetaxel induced by miR-129 is dependent on CP110 inhibition, we transfected CP110 into MDA-MB-231/Doc, MDA-MB-231-miR-129 and MCF-7-miR-129 cells. Then the transfected cells were exposed to various concentrations of docetaxel for 48 hrs, and cell viability was assessed via MTT assay. As shown in Fig. 3E–G, CP110 transfection could restore the sensitivity of MDA-MB-231/Doc, MDA-MB-231-miR-129, and MCF-7-miR-129 cells to docetaxel. The transfection efficiency was detected by western blot analysis. In addition, we confirmed a 10 nM docetaxel concentration-dependent curve based on the cell viability analysis of MDA-MB-231-miR-129 and MCF-7-miR-129 cells with or without CP110 overexpression on different time points. Our results demonstrated that CP110 could significantly re-sensitize breast cancer cells to docetaxel (Fig. 3H).

### MiR-129 promoted, while CP110 inhibited, DNA synthesis and cell proliferation of breast cancer cells upon docetaxel treatment

**Figure 4:**
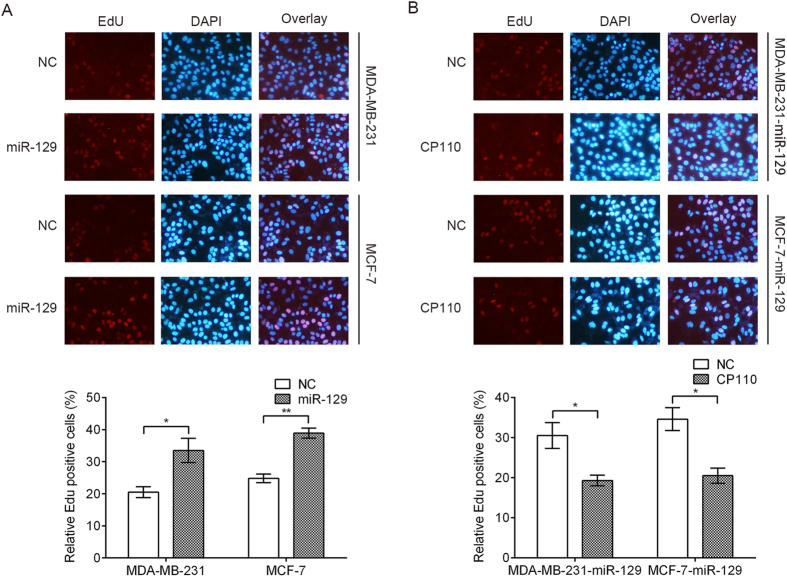
EdU staining for evaluation of the influences of miR-129 and CP110 on the proliferation of breast cancer cells. Cells were exposed to docetaxel (10 nm) for 48 hrs and subjected to EdU incorporation assays. The new generation cells were detected via EdU (red). DAPI stained nuclei in blue. Merged view of EdU (red) and DAPI (blue) showing the overlap. (**A**) Proliferative ability data for MDA-MB-231 and MCF-7 cells transfected with negative control (NC) and miR-129. (**B**) Proliferative ability data for MDA-MB-231-miR-129 and MCF-7-miR-129 cells transfected with negative control (NC) and CP110. Each bar represents mean ± SD. p values were calculated using a Student t-test (*p < 0.05, **p < 0.01).

We further determined the effect of miR-129 and CP110 on DNA synthesis and cell proliferation using an EdU assay. We transfected MDA-MB-231 and MCF-7 cells with miR-129 or NC (negative control), then treated them with 10 nM docetaxel for 48 hrs. Compared to the NC group, the number of EdU-positive cells significantly increased upon miR-129 transfection (Fig. 4A), suggesting that miR-129 increases the DNA synthesis upon docetaxel treatment. Simultaneously, we transfected MDA-MB-231-miR-129 and MCF-7-miR-129 cells with CP110 or control respectively then treated them with 10 nM docetaxel for 48 hrs. Compared to the control group, the number of EdU-positive cells significantly decreased upon CP110 overexpression (Fig. 4B), suggesting that CP110 inhibited the DNA synthesis upon docetaxel treatment.

### MiR-129-3p reduced G2/M arrest and apoptosis of breast cancer cells following docetaxel exposure

**Figure 5:**
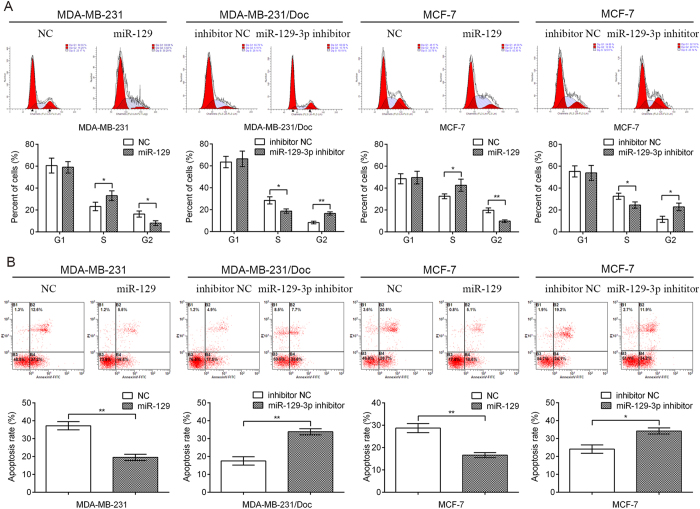
MiR-129-3p promote cell proliferation and inhibited apoptosis in MDA-MB-231, MDA-MB-231/Doc and MCF-7 cells. MDA-MB-231, MDA-MB-231/Doc and MCF-7 cells transfected with miR-129, miR-129-3p inhibitor or both were treated with 10 nM docetaxel for 48 hrs. Flow cytometric analysis of cell cycle (**A**) and apoptosis (**B**) indicated that miR-129-3p down-regulation significantly induced cell apoptosis and cell cycle arrest in G2/M phase, vice versa. Data are presented as the mean ± SD. *p < 0.05, **p < 0.01 by Student’s t-test.

We examined docetaxel-induced cell cycle arrest and apoptosis in MDA-MB-231 cells, MDA-MB-231/Doc cells, and MCF-7 cells following miR-129 or miR-129-3p inhibitor transfection. As shown in Fig. 5A, miR-129 overexpression resulted in a decreased percentage of MDA-MB-231 cells in G2/M phase, whereas down-regulation of miR-129-3p triggered MDA-MB-231/Doc cell cycle arrest in G2/M phase. In addition, a significant decrease in apoptosis was observed in MDA-MB-231 cells transfected with miR-129 after docetaxel treatment compared with NC cells, whereas a marked increase in apoptosis was found in MDA-MB-231/Doc cells transfected with miR-129-3p inhibitor after docetaxel treatment compared with control transfected cells (Fig. 5B). Furthermore, similar results were obtained in MCF-7 cells (Fig. 5A,B). These data suggested that miR-129-3p decreased docetaxel sensitivity mainly through promoting cell growth and inhibiting apoptosis in breast cancer cells.

### CP110 induced G2/M arrest and apoptosis of breast cancer cells following docetaxel exposure

**Figure 6:**
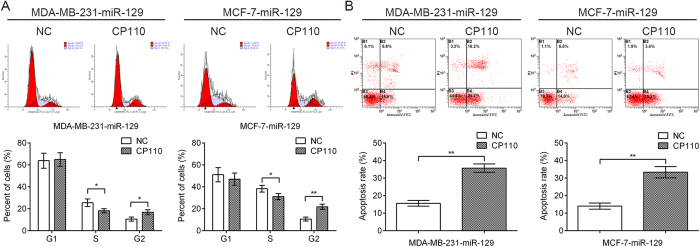
CP110 inhibited cell proliferation and promoted apoptosis in MDA-MB-231-miR-129 and MCF-7-miR-129 cells. MDA-MB-231-miR-129 and MCF-7-miR-129 cells transfected with negative control (NC) or CP110 were treated with 10 nM docetaxel for 48 hrs. Flow cytometric analysis of cell cycle (**A**) and apoptosis (**B**) indicated that CP110 overexpression significantly induced cell apoptosis and cell cycle arrest in G2/M phase. Data are represented as the mean ± SD of each group. *p < 0.05, **p < 0.01 by Student’s t-test.

To analyze the effect of CP110 on the cell cycle and apoptosis following docetaxel treatment, we examined docetaxel-induced cell cycle arrest and apoptosis in MDA-MB-231-miR-129 and MCF-7-miR-129 cells following CP110 or NC transfection. CP110 overexpression resulted in an increased percentage of MDA-MB-231-miR-129 and MCF-7-miR-129 cells in G2/M phase (Fig. 6A). In addition, CP110 could dramatically increase the apoptosis rates of MDA-MB-231-miR-129 and MCF-7-miR-129 cells with docetaxel treatment (Fig. 6B).

### MiR-129 overexpression induces docetaxel resistance of breast cancer cells in a xenograft model

**Figure 7:**
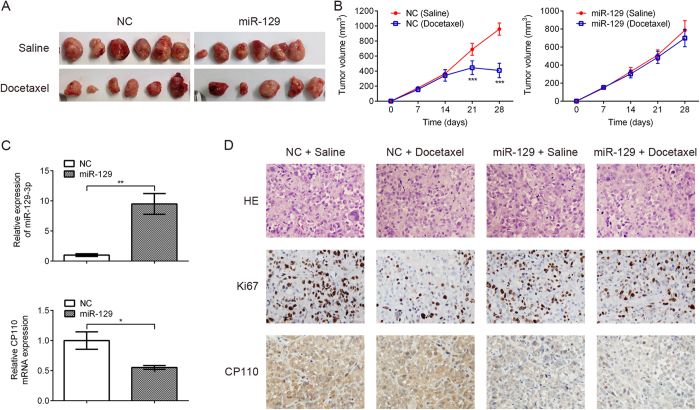
MiR-129 overexpression enhances docetaxel resistance *in vivo*. (**A**) Representative pictures of xenograft tumors are shown. (**B**) Growth curves of tumors derived from miR-129 and NC-transfected MDA-MB-231 cells. (**C**) Quantitative real-time PCR analysis showing that miR-129-3p was up-regulated and CP110 was down-regulated in transplanted tumor tissues inoculated by miR-129-overexpressing MDA-MB-231 cells. (**D**) HE-stained, Ki67-stained and CP110-stained sections of the transplanted tumors are shown. Data are presented as the mean ± SD (n = 3). *p < 0.05, **p < 0.01, ***p < 0.001 by Student’s t-test.

To further confirm the role of miR-129 in docetaxel resistance, we stably transfected cells with miR-129 using lentiviral vectors. We injected 5 × 10^6^ cells stably expressing miR-129 into both flanks of nude mice and initiated docetaxel therapy 2 weeks later at a dose of 10 mg/kg once a week for 2 weeks. We examined human miR-129 expression efficiency using quantitative real-time PCR of tumor tissues implanted in nude mice. In addition, we quantified CP110 and Ki67 expression levels in tumor xenografts using immunostaining analysis. As shown in Fig. 7A,B, tumor growth was significantly inhibited after docetaxel treatment in the negative control group compared to the miR-129 overexpression group. As expected, miR-129 overexpression attenuated docetaxel-induced cell death, and miR-129-overexpressing MDA-MB-231 cells showed increased cell growth *in vivo* relative to negative control cells. As shown in Fig. 7C, quantitative real-time PCR analysis revealed that CP110 mRNA decreased due to increased miR-129 expression. Moreover, immunohistochemical staining showed that positive CP110 expression in tumors formed from miR-129-transfected MDA-MB-231 cells was decreased in comparison with that in NC tumors (Fig. 7D). These results indicate that increased miR-129 expression promoted *in vivo* cell proliferation, consistent with our *in vitro* findings.

## Discussion

Because docetaxel is often used in the treatment of many types of cancer, including breast cancer, the development of chemosensitization strategies will have important clinical implications. Increasing evidence supports the view that miRNAs are key modulators of drug resistance and consequently, that modulation of their activity could be a new treatment strategy. This possibility is mainly attributed to the ability of miRNAs to regulate gene expression and participate in gene regulatory networks by sequence-specific binding to their target mRNAs. The multidrug resistance 1 (MDR1) protein, a crucial factor in drug resistance, has been linked to docetaxel resistance and disease progression in breast cancer^37^. In our study, a subset of miRNAs (miR-129, miR-186, miR-214, miR-223 and miR-374) was predicted to interact with the 3′ UTR of MDR1 mRNA by bioinformatics analysis. We evaluated the effects of these five miRNAs on MDR1 expression and docetaxel resistance in breast cancer cells. Interestingly, we observed that miR-129 overexpression unexpectedly increased the docetaxel resistance of breast cancer cells, whereas overexpression of the other four miRNAs had no effect, in contrast with our original hypothesis. A possible explanation for this finding is that only miR-129-5p is predicted to interact with the 3′ UTR of MDR1 mRNA, and miR-129-3p is the main mature miRNA formed from the miR-129 precursor in breast cancer cells. Consequently, we will further study the novel biological function and molecular mechanism of miR-129-3p to gain insights to aid in the development of an effective strategy for reversing the docetaxel resistance of breast cancer.

MiR-129 expression has been investigated in many human cancers, including gastric cancer, esophageal cancer, bladder cancer, colorectal cancer, endometrial cancer, and hepatocellular carcinoma^27^,^38^,^39^,^40^,^41^,^42^. In addition, miR-129 expression has been shown to correlate with patient survival^23^,^27^,^29^,^40^. Regarding the association of miR-129 and drug resistance, it has recently been reported that miR-129 promotes apoptosis and enhances chemosensitivity to 5-fluorouracil of colorectal cancer cells by negatively regulating BCL2^28^. However, in this study, we found that miR-129-3p inhibited docetaxel-induced apoptosis of breast cancer cells by down-regulation of the CP110 protein. According to miRNA target databases, one miRNA can regulate the expression of multiple target genes, while one gene may be targeted by many miRNAs. A miR-129-induced target molecule alteration was deemed potentially responsible for this phenomenon. A possible explanation for the lack of effects on BCL2 expression observed in this study could be that docetaxel triggers apoptosis of breast cancer cells through a pathway that does not involve BCL2.

In this study, we found that miR-129-3p expression was up-regulated in the docetaxel-resistant human breast cancer cell line MDA-MB-231/Doc compared to its parental cell line MDA-MB-231. We identified miR-129-3p as a miRNA that can enhance docetaxel chemotherapy resistance in breast cancer cells. In addition, our data showed that miR-129-3p had no effect on cell proliferation, DNA synthesis, cell cycle or apoptosis in breast cancer cells without drug treatment (Supplementary Fig. S1 and S2). Our findings suggest that miR-129-3p overexpression increased breast cancer cell resistance to docetaxel mainly through down-regulating CP110 expression *in vitro* and *in vivo*. Importantly, miR-129-3p repression sensitized breast cancer cells to docetaxel by delaying G2/M progression and increased apoptosis. To the best of our knowledge, our study is the first to show the association between miR-129-3p expression and docetaxel chemoresistance in human breast cancer.

In search of the molecular basis for the phenotype of miR-129 overexpression, CP110 was identified as a direct target of miR-129. Previous studies found that miR-129-3p controls cilia assembly by regulating CP110 and actin dynamics, suggesting that miR-129-3p promotes docetaxel resistance via the regulation of microtubule and centrosome formation^34^. We found that CP110 was down-regulated in the docetaxel-resistant human breast cancer cell line MDA-MB-231/Doc compared to the parental cells. Furthermore, CP110 shRNA-transfected MDA-MB-231 cells presented much lower sensitivity to docetaxel than the negative control groups, with a greater decrease in CP110 mRNA and protein expression. We also noticed an inverse correlation between miR-129-3p and CP110, and confirmed that CP110 is a direct miR-129-3p target. Cyclin-dependent kinases (CDKs) play essential roles in regulating the cell division cycle, apoptosis, transcription and differentiation^43^. CP110 is an integral centrosomal component and is phosphorylated by CDKs to drive centrosomal and cell cycles^35^. Most importantly, CP110 gene expression is induced at the G1-to-S transition, reaching peak levels in S phase, then decreasing during mitosis. In docetaxel-treated cells, centrosome organization is disrupted, affecting late S phase, G2 and M phases, which results in cell cycle arrest and cell death^44^. We analyzed the effects of miR-129-3p expression on docetaxel-induced cell death and found that changes in miR-129-3p expression affected apoptosis. However, further research is required to fully understand the detailed mechanism. *In vivo* experiments suggest that miR-129 overexpression significantly decreases docetaxel-mediated growth inhibition of xenografts in mice. Taken together, our data suggest that miR-129-3p is associated with docetaxel resistance in breast cancer cells and that CP110 is a direct miR-129-3p target.

In summary, our findings present the first evidence that miR-129-3p may be involved in the development of docetaxel resistance in breast cancer cell lines. MiR-129-3p may modulate breast cancer cell sensitivity to docetaxel, mainly through CP110 repression. Our data provide a novel insight into the mechanisms of docetaxel resistance, and manipulation of miR-129-3p expression may be a promising therapeutic strategy for overcoming docetaxel resistance in breast cancer.

## Methods

### Cell lines and cell culture

The human breast cancer cell lines MDA-MB-231 and MCF-7 were purchased from the American Type Culture Collection (Manassas, VA, USA) and were confirmed by STR analysis (Supplementary Figs S3 and S4 online). The docetaxel-resistant MDA-MB-231 cell line (MDA-MB-231/Doc) was established and preserved in a 10 nM final concentration of docetaxel in our laboratory for 8 months. All cells were cultured in Dulbecco’s modified essential medium (DMEM) containing 10% fetal bovine serum and ampicillin and streptomycin at 37 °C in a humidified atmosphere of 95% air/5% CO_2_.

### Transfection

The miR-129-overexpressing plasmid containing full-length genomic miR-129 (plenti6.3/miR-129) DNA and the mock plenti6.3-negative control (plenti6.3/NC) were generated in our laboratory. The miR-129-3p inhibitor, miR-129-3p inhibitor negative control, CP110 short hairpin RNA (shRNA/CP110), and non-specific control shRNA (shRNA/NC) were chemically synthesized by GenePharma (Shanghai, China). The sequence for miR-129 gene-expressing plasmid were as follows: miR-129 forward, 5′-GGGGGATCCATTACAGCTGGGATTCCTGTTGCC-3′ and reverse 5′-GGGCTCGAGGTGGAGTTCTCTCACTTGACATTGC-3′; the sequence of the mock plenti6.3-negative control were as follows: sense, 5′-UUCUCCGAACGUGUCACGUTT-3′, and anti-sense, 5′-ACGUGACACGUUCGGAGAATT-3′; miR-129-3p mimics, 5′-AAGCCCUUACCCCAAAAAGCAU-3′ and 5′-GCUUUUUGGGGUAAGGUCUUUU-3′; miR-129-5p mimics, 5′-CUUUUUGCGGUCUGGGCUUGC-3′ and 5′-AAGCCCAGACCGCAAAAAGUU-3′. The miR-129-3p inhibitor sequence was 5′-AUGCUUUUUGGGGUAAGGGCUU-3′. The miR-129-3p inhibitor negative control sequence was 5′-CAGUACUUUUGUGUAGUACAA-3′. The sequences of the shRNAs were as follows: shRNA/CP110 sense, 5′-AAGCAGCAUGAGUAUGCCAGUTT-3′, and antisense, 5′-ACUGGCAUACUCAUGCUGCUUTT-3′; shRNA/NC sense, 5′-UUCUCCGAACGUGUCACGUTT-3′, and antisense, 5′-ACGUGACACGUUCGGAGAATT-3′. All plasmid DNA was extracted using a Plasmid Mini Kit (Qiagen, Hilden, Germany). Cells were plated into 6-well plates and transfected with Lipofectamine 2000 (Invitrogen, Carlsbad, CA, USA) according to the manufacturer’s protocol. For stably transfected cells, cells were transfected with lentivirus at 80–90% confluence in six-well plates. The cells were infected with recombinant lentivirus in the presence of 8 μg/mL polybrene (Sigma, St. Louis, MO, USA).

### MTT assay

Cell survival was measured by 3-(4, 5-dimethylthiazol-2-yl)-2, 5-diphenyltetrazolium bromide (MTT) assay. Untransfected or transfected cells were re-seeded into 96-well plates and incubated at 37 °C in humidified 5% CO_2_ for 48 hrs. Serially diluted docetaxel was added, and the cells were incubated for an additional 48 hrs. The absorbance at 490 nm (A490) of each well was measured on a spectrophotometer. Three independent experiments were performed in quadruplicate.

### Colony formation assay

A total of 1 × 10^5^ cells were seeded into each well of a six-well plate and treated with 10 nM docetaxel for 48 hrs. After 14 days, cells were fixed with methanol and stained with 0.1% crystal violet. The samples were photographed, and visible colonies were manually counted. Colonies containing at least 50 cells were counted.

### Quantitative real-time PCR

Total RNA isolation from cell lines was performed using Trizol (Invitrogen, Carlsbad, CA, USA). Reverse transcription (RT) was performed with the TaqMan MicroRNA Reverse Transcription kit (Applied Biosystems, Foster City, CA, USA). To analyze coding gene expression, cDNA was synthesized using Superscript III reverse transcriptase (Invitrogen, Carlsbad, CA, USA) according to the manufacturer’s instructions. Quantitative real-time PCR was performed on an ABI 7500 real-time system (Applied Biosystems, Foster City, CA, USA) according to the manufacturer’s protocol. Data were analyzed according to the comparative Ct method^45^. GAPDH or U6 was used as an endogenous control. All reactions were performed in triplicate, and at least 3 independent experiments were performed to generate each data set. Polymerase chain reaction (PCR) primers were as follows: miR-129-3p, 5′-AAGCCCTTACCCCAAAAAGTAT-3′; miR-129-5p, 5′-CTTTTTGCGGTCTGGGCTTGC-3′; U6 sense, 5′-GCTTCGGCAGCACATATACTAAAAT-3′, and reverse, 5′-CGCTTCACGAATTTGCGTGTCAT-3′; CP110 sense, 5′-AGACGCAGTCTGAGAGGTAGT-3′, and reverse, 5′-CAGTGTTTGCCTGTCAACTGG-3′; GAPDH sense, 5′-GGTGAAGGTCGGAGTCAACG-3′, and reverse, 5′-CAAAGTTGTCATGGATGHACC-3′.

### Western blotting

Cells were harvested and homogenized with lysis buffer, and western immunoblotting was performed using standard procedures. Total protein was separated on 12% SDS-PAGE gels and transferred to PVDF membrane (Millipore, Bedford, MA, USA). The membrane was blocked with 5% nonfat dry milk in TBS, then probed with a CP110-specific primary antibody (1:1000, ProteinTech Group, Chicago, IL, USA) or β-tubulin (1:1000, ProteinTech Group, Chicago, IL, USA) which was used as an internal control (see Supplementary Fig. S5 online). After washing, horseradish peroxidase-conjugated anti-rabbit IgG (1:2000, Santa Cruz Biotechnology, Santa Cruz, CA, USA) was used as a secondary antibody and incubated for 1 hr at room temperature. Immunoreactive proteins were detected using the ECL system (Millipore, Bedford, MA, USA).

### Flow cytometry for apoptosis and cell cycle analysis

Cells transfected with miR-129, miR-129-3p inhibitor or NC were treated with 10 nM docetaxel for 48 hrs and harvested. For the cell cycle assay, cells were washed with ice-cold phosphate-buffered saline (PBS) and fixed with ice-cold 70% ethanol overnight at −20 °C. Fixed cells were rehydrated in PBS for 20 min and subjected to PI/RNase staining. Flow cytometry was performed using a FACScan instrument (Becton Dickinson, Franklin Lakes, NJ, USA), and analysis was performed with CellQuest software (Becton Dickinson, Franklin Lakes, NJ, USA). For cell apoptosis analysis, apoptotic cells were detected with the Annexin V-FITC Apoptosis Detection Kit (Oncogene Research Products, Boston, MA, USA) according to the manufacturer’s instructions and analyzed using flow cytometry (Becton Dickinson, Franklin Lakes, NJ, USA).

### Luciferase reporter assay

The miR-129-3p or miR-129-5p response element (wild type or mutant) was cloned into the 3′ UTR of CP110 into the pMIRREPORT plasmid downstream of the luciferase reporter gene. A luciferase assay kit (Promega, Madison, WI, USA) was used according to the manufacturer’s protocol. Firefly luciferase activity was normalized to Renilla luciferase activity for each transfected well.

### EdU proliferation assay

To measure cell proliferation, an EdU (5-ethynyl-2′-deoxyuridine) proliferation assay was performed as described previously^46^. MDA-MB-231 and MCF-7 cells transfected with miR-129 or NC were plated in 24-well plates at a density of 5 × 10^4^ cells/well, then treated with 10 nM docetaxel for 48 hrs. Cells were washed with PBS, then incubated in serum-free DMEM containing 10 μmol/L EdU (RiboBio, Guangzhou, China) for 2 hrs. Cells were fixed, then underwent Apollo staining and DNA staining, according to the manufacturer’s instructions to detect the number of cycling cells during the EdU treatment. The cells were imaged using fluorescence microscopy, and the number of proliferating cells was averaged to calculate the labeling index.

### Nude mouse model

The murine studies were conducted in accordance with the Institutional Animal Care and Use Committee (IACUC) guidelines and were approved by the Institutional Animal Ethics Committee of the Fourth Military Medical University. Female BALB/C nude mice aged 4–6 weeks old were purchased from Shanghai Laboratory Animal Center (SLAC, Shanghai, China) and housed within a dedicated SPF facility at the Laboratory Animal Center of the Fourth Military Medical University (NO.13002, see Supplementary Fig. S6 online). Animal protocols were approved by the Institutional Animal Ethics Committee of the Fourth Military Medical University. MDA-MB-231 cells (5 × 10^6^) were transfected with NC or miR-129 and subcutaneously injected into both flanks of nude mice. Tumor size was measured every 2 days using a slide caliper, and tumor volume was calculated using the formula V = length × width^2^/2. After 14 days, docetaxel therapy was initiated via intraperitoneal injection at a dose of 10 mg/kg, twice per week. The mice were humanely killed on day 28, and subcutaneous tumors were surgically excised, weighed, photographed, sectioned, and stained with hematoxylin-eosin. CP110 and miR-129-3p expression levels in tumors were measured with quantitative real-time PCR. Cryosections were stained with hematoxylin and eosin and used for immunohistochemistry.

### Immunohistochemistry

Immunohistochemical staining of Ki67 and CP110 was performed as described previously^47^. Briefly, paraffin-embedded 4 μm-thick sections were deparaffinized, heated in citrate buffer (0.01 M), treated with 0.3% H_2_O_2_ (v/v), and re-hydrated. After blocking, rabbit polyclonal anti-CP110 (1:100, ProteinTech Group, Chicago, IL, USA) and anti-Ki67 (1:50, ProteinTech Group, Chicago, IL, USA) primary antibodies incubated overnight at 4 °C. After rinsing in PBS, slides were incubated for 25 min at room temperature with biotinylated goat-anti-rabbit immunoglobulin followed by incubation with peroxidase-conjugated streptavidin for 20 min and fresh 0.05% 3, 3′-diaminobenzidine (DAB). Slides were counterstained with Mayer’s hematoxylin and mounted with crystal mount. The methods were carried out in accordance with the approved guidelines and all experimental protocol were approved by a named institutional and/or licensing committee.

### Statistical analysis

GraphPad Prism software (version 5.0) was used for statistical analysis. All data are expressed as the mean ± SD. The difference between the means was analyzed with Student’s t-test or Fisher’s exact test. Statistical significance was considered p < 0.05.

## Additional Information

**How to cite this article**: Zhang, Y. *et al.* MiR-129-3p promotes docetaxel resistance of breast cancer cells via CP110 inhibition. *Sci. Rep.* **5**, 15424; doi: 10.1038/srep15424 (2015).

## Supplementary Material

Supplementary Information
