# Supplementary material for: MiR-129-3p promotes docetaxel resistance of breast cancer cells via CP110 inhibition
Source: Sci Rep. 2015 Oct 21;5:15424. doi: 10.1038/srep15424 (PMC4614026; doi:10.1038/srep15424)
Supplement: Supplementary Information [file srep15424-s1.doc]

## Supplementary Information

**MiR-129-3p promotes docetaxel resistance of breast cancer cells via CP110 inhibition**

Yuan Zhang^1, 2^,**^§^**, Yu Wang^1^,**^§^**, Yifang Wei^2^, Mengyang Li^2^, Shentong Yu^2^, Mingxiang Ye^3^, Hongmei Zhang^1^, Suning Chen^4^*, Wenchao Liu^1^*, Jian Zhang^2^*


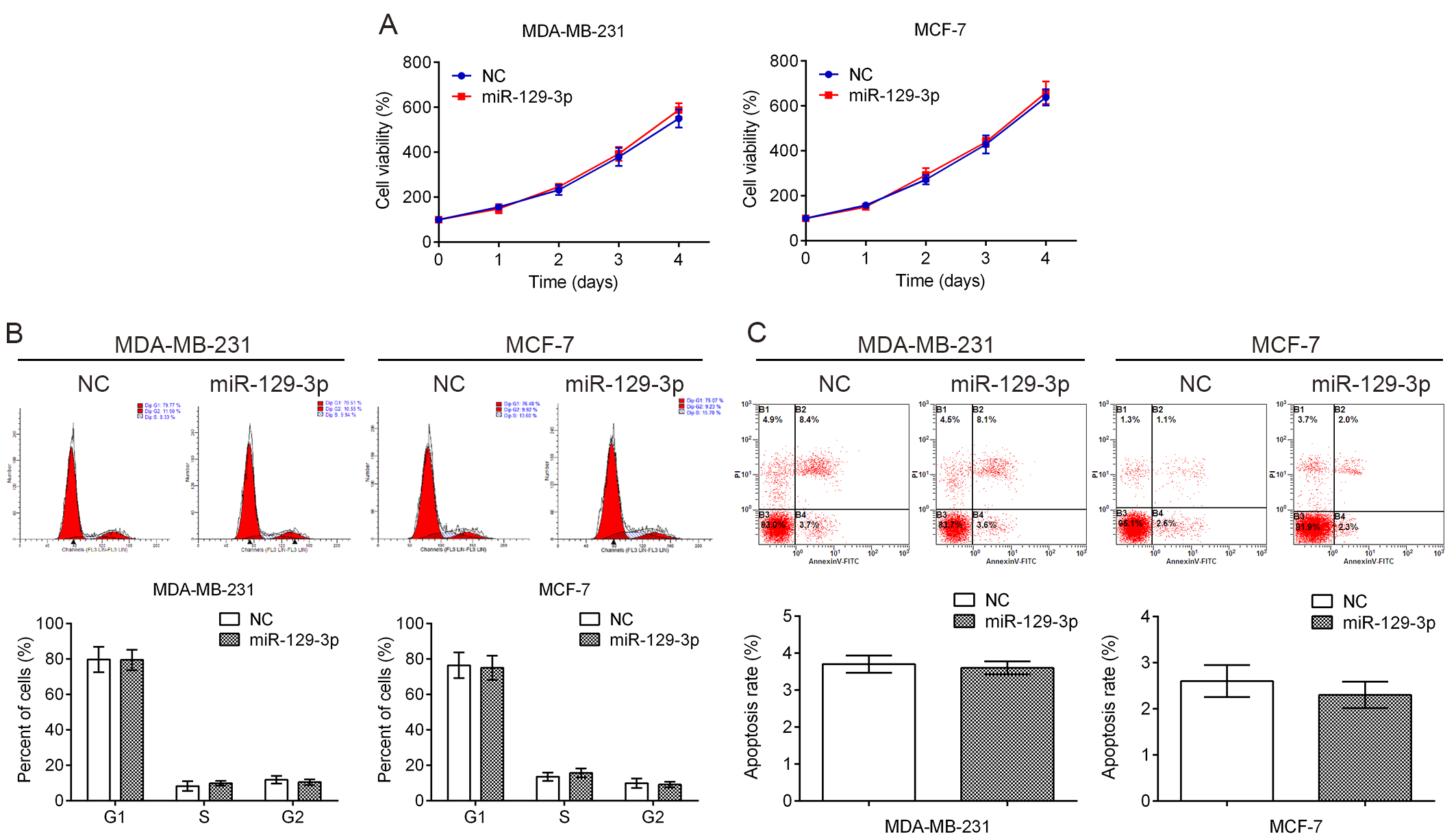


**Supplementary Figure 1.** In the absence of docetaxel treatment, MDA-MB-231 cells and MCF-7 cells were transfected with miR-129-3p mimics or NC. The effects of miR-129-3p on cell proliferation (A), cell cycle (B) and apoptosis (C) were assessed 48 hrs after transfection, respectively. The data are presented as the means ± SD from triplicate analyses. There are no significant differences between miR-129-3p-transfected group and NC group.


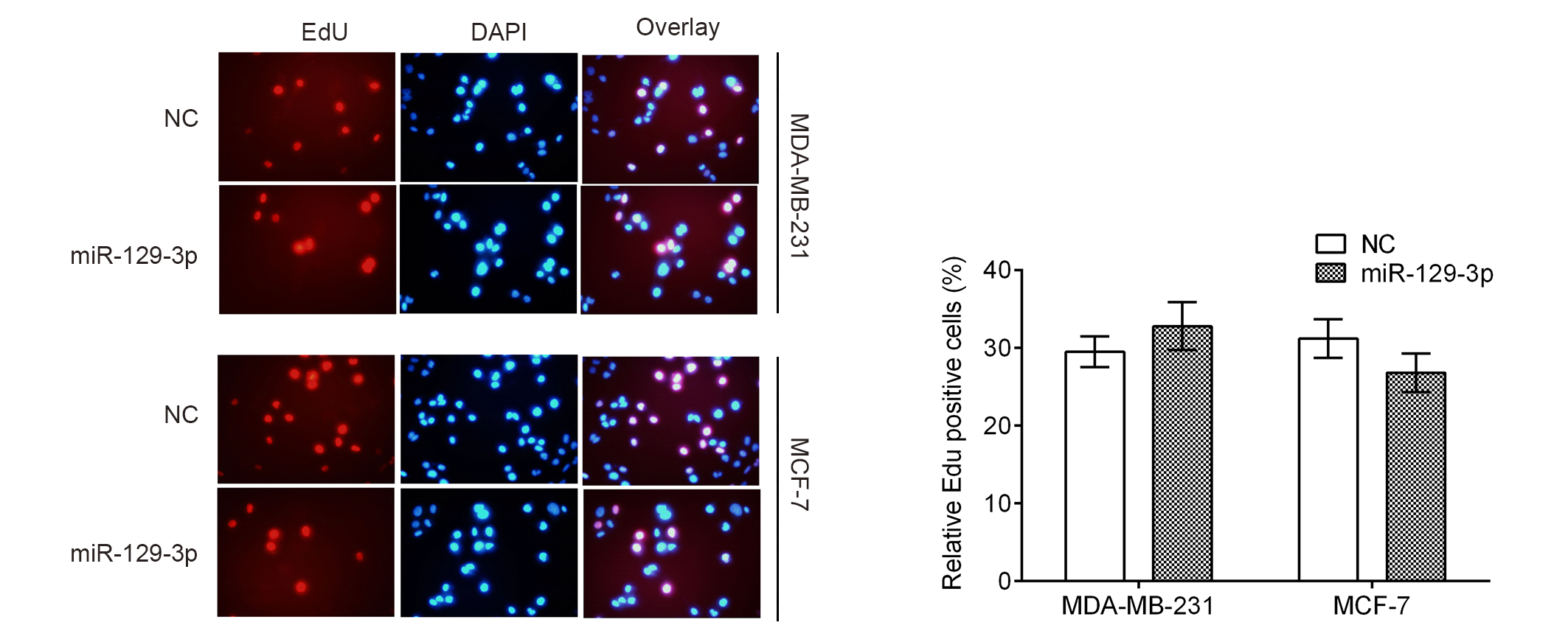


**Supplementary Figure 2.** In the absence of docetaxel treatment, MDA-MB-231 cells and MCF-7 cells were transfected with miR-129-3p mimics and NC. EdU analysis of miR-129-3p on the proliferation of breast cancer cells was performed 48 hrs after transfection. The data are presented as the means ± SD from triplicate analyses. There are no significant differences between miR-129-3p-transfected group and NC group.


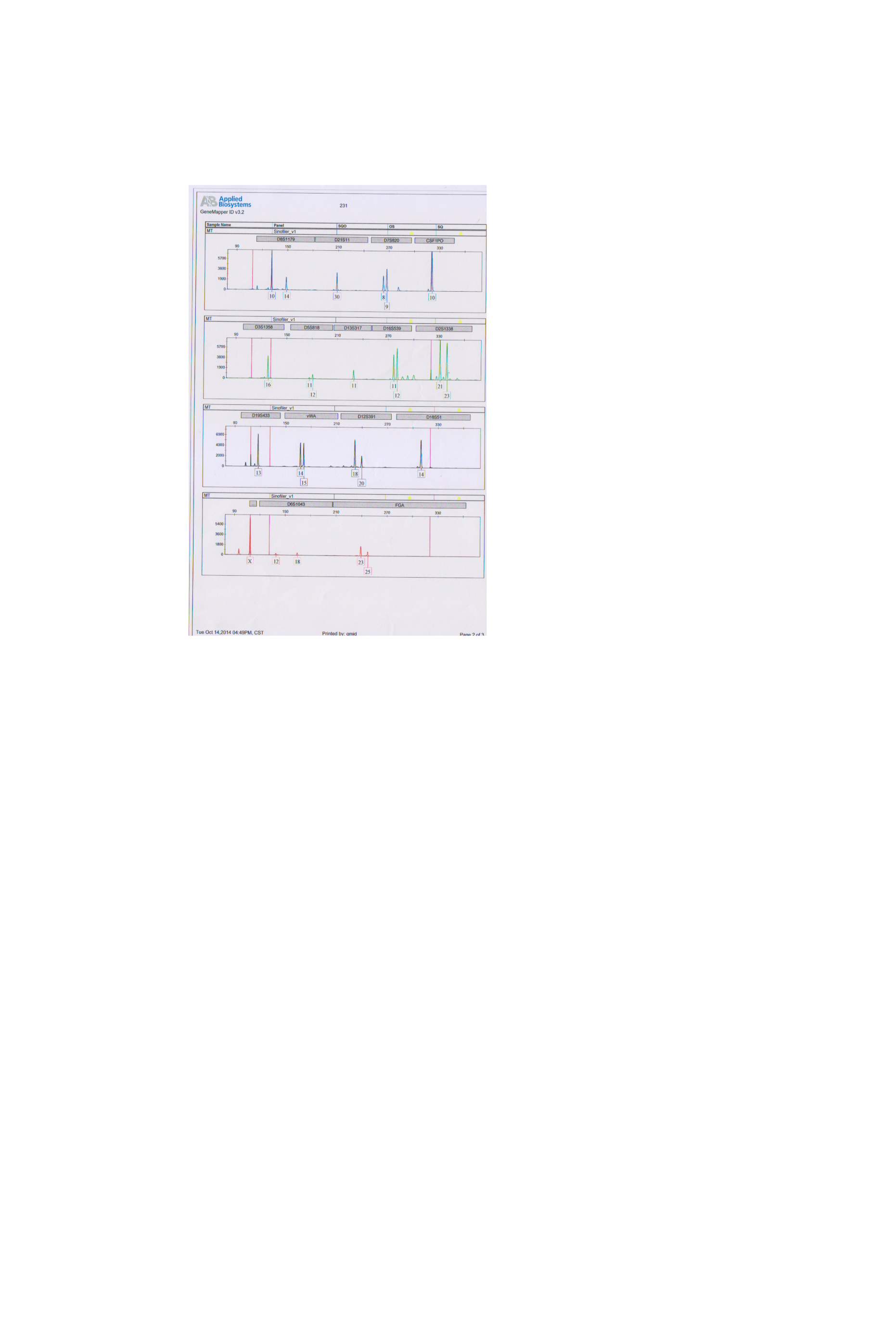


**Supplementary Figure 3.** The MCF-7 cells were authenticated by short tandem repeat (STR) analysis.


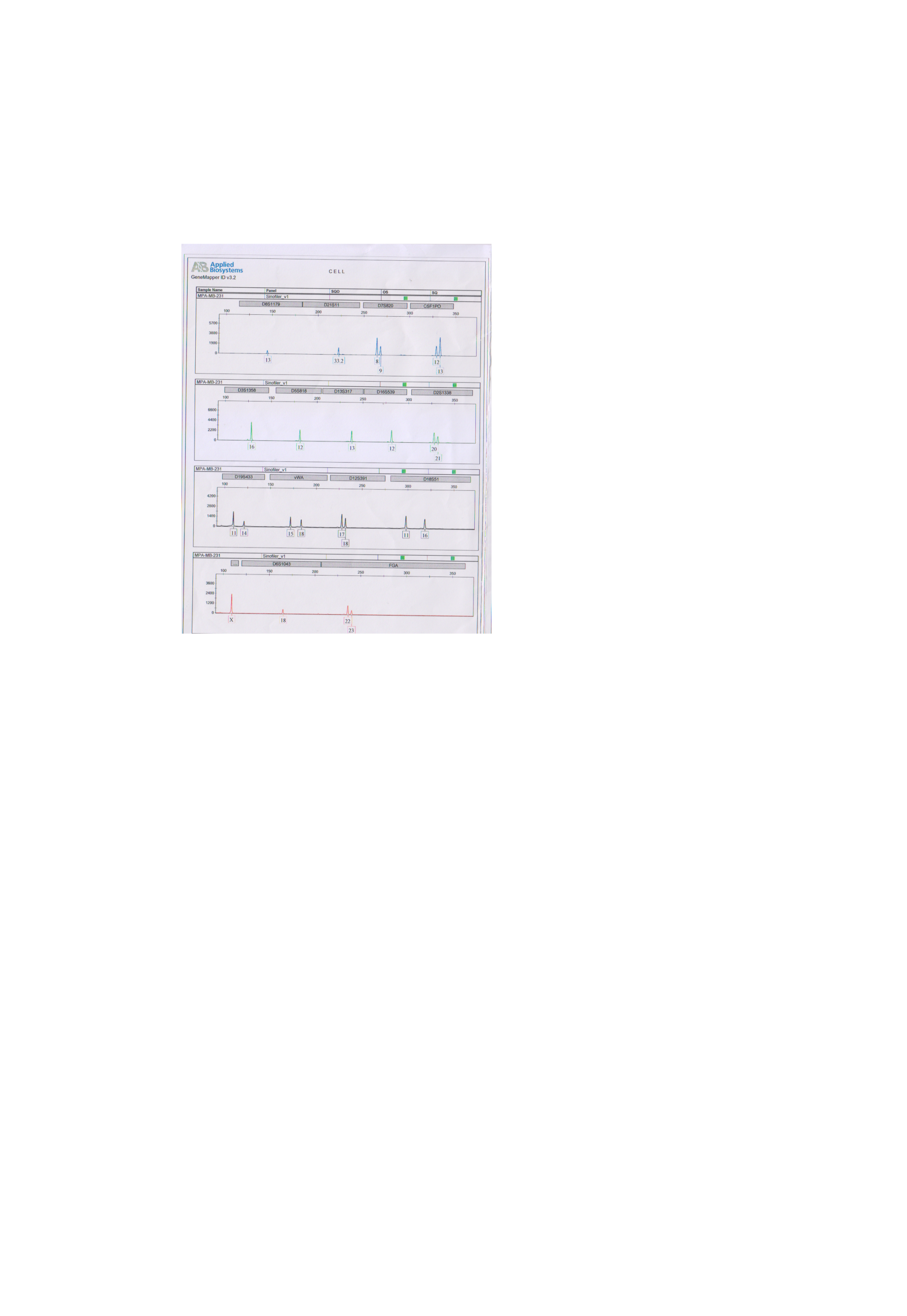


**Supplementary Figure 4.** The MDA-MB-231 cells were authenticated by short tandem repeat (STR) analysis.


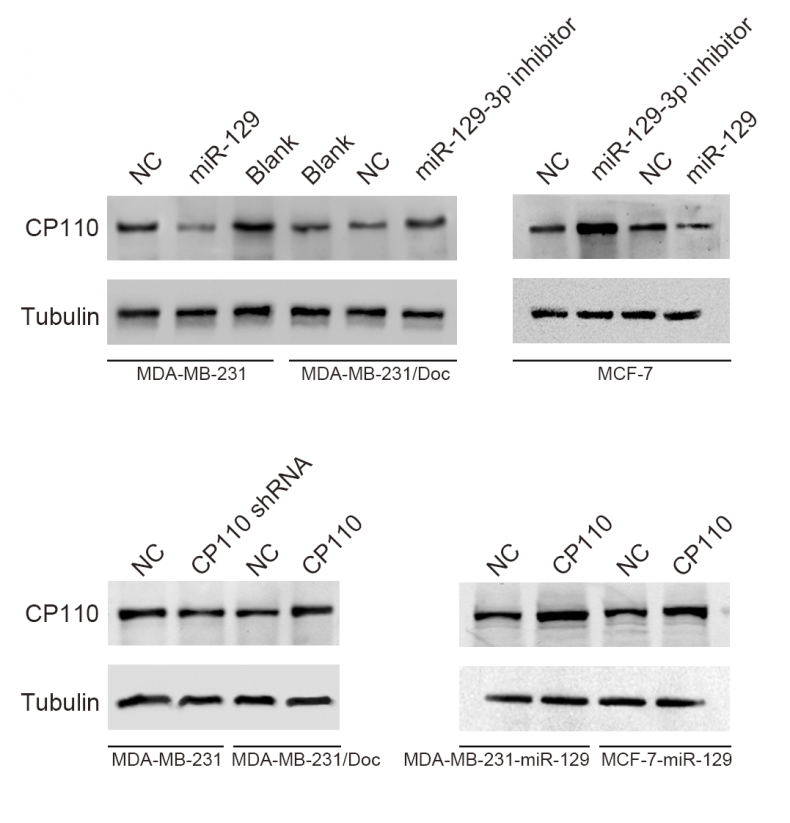


**Supplementary Figure 5.** Western blot analysis showed that miR-129 overexpression inhibited CP110 protein expression and downregulation of miR-129-3p facilitated CP110 protein expression in breast cancer cells. Western blot was applied to validate the efficiency of overexpressed and intervened CP110 protein in breast cancer cells.


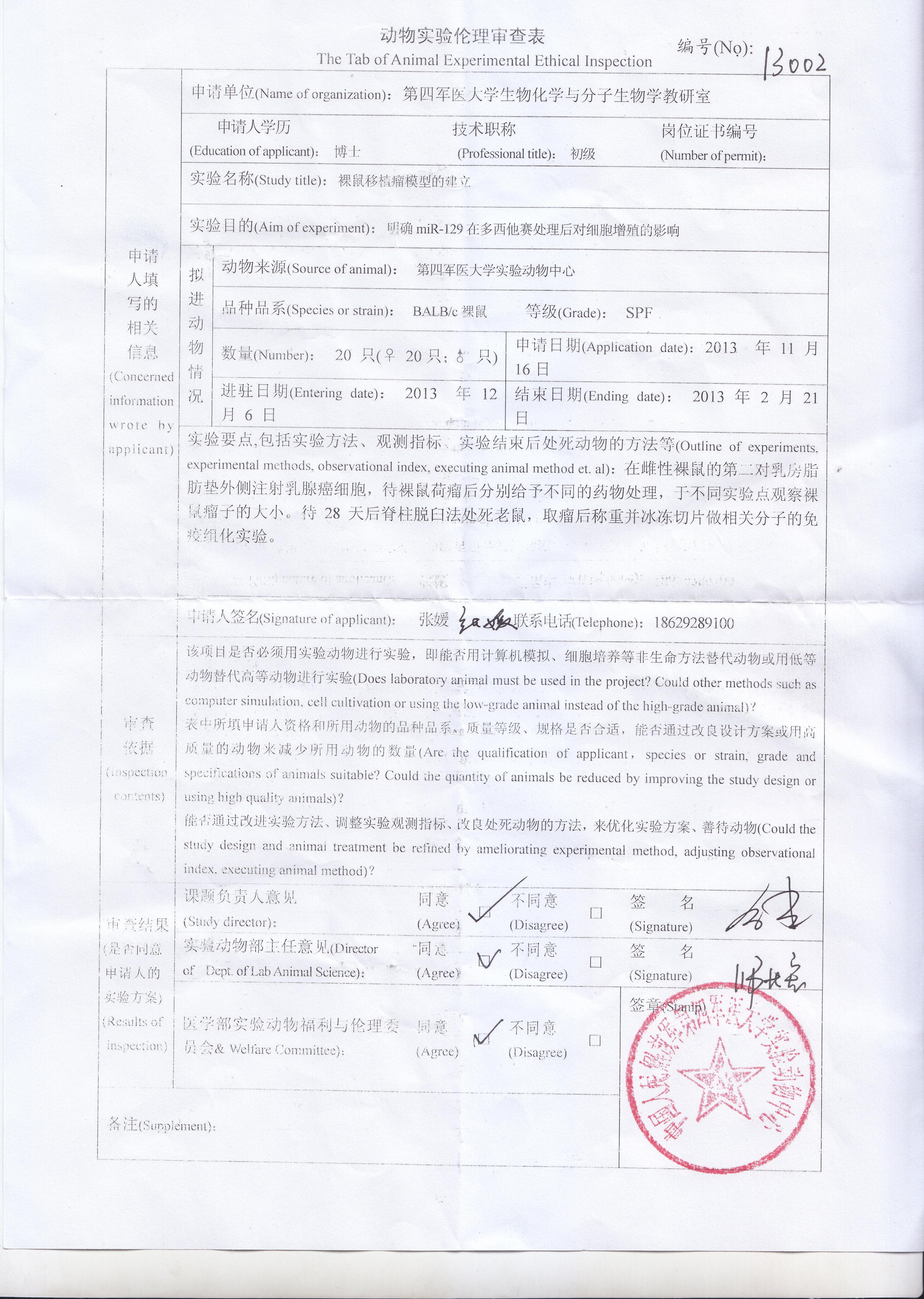


**Supplementary Figure 6.** The tab of animal experimental ethical inspection.
